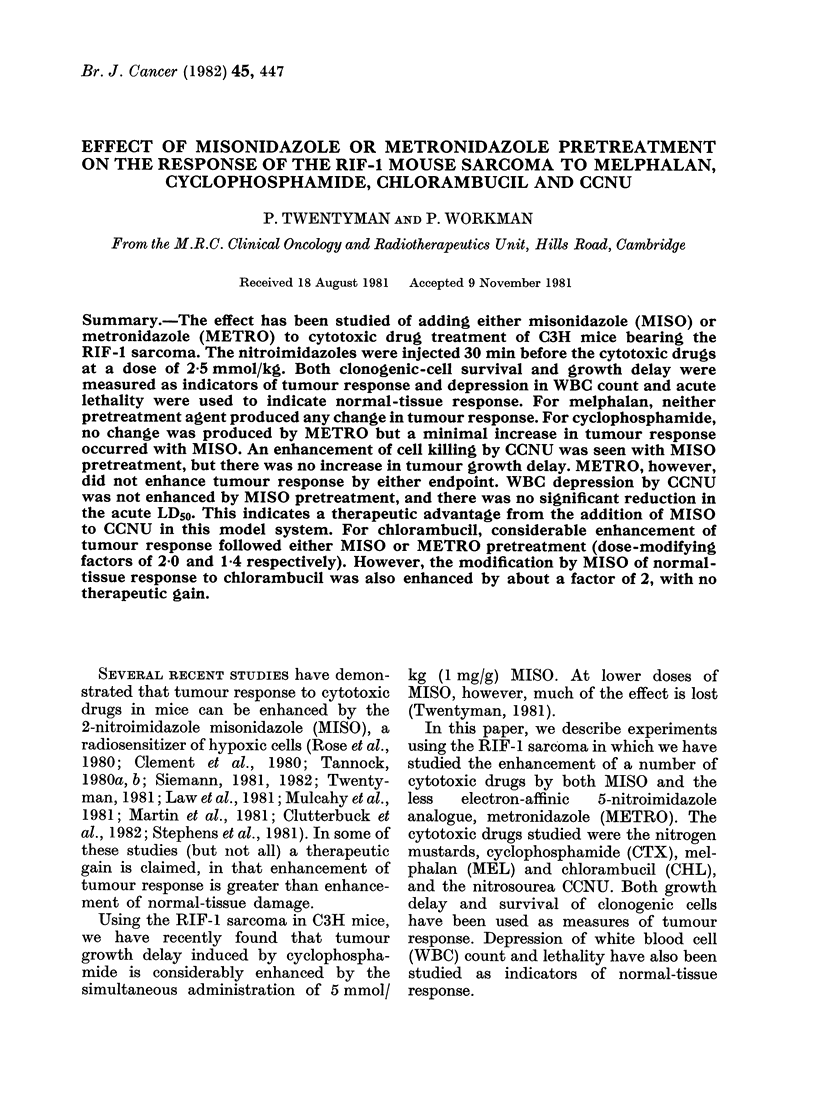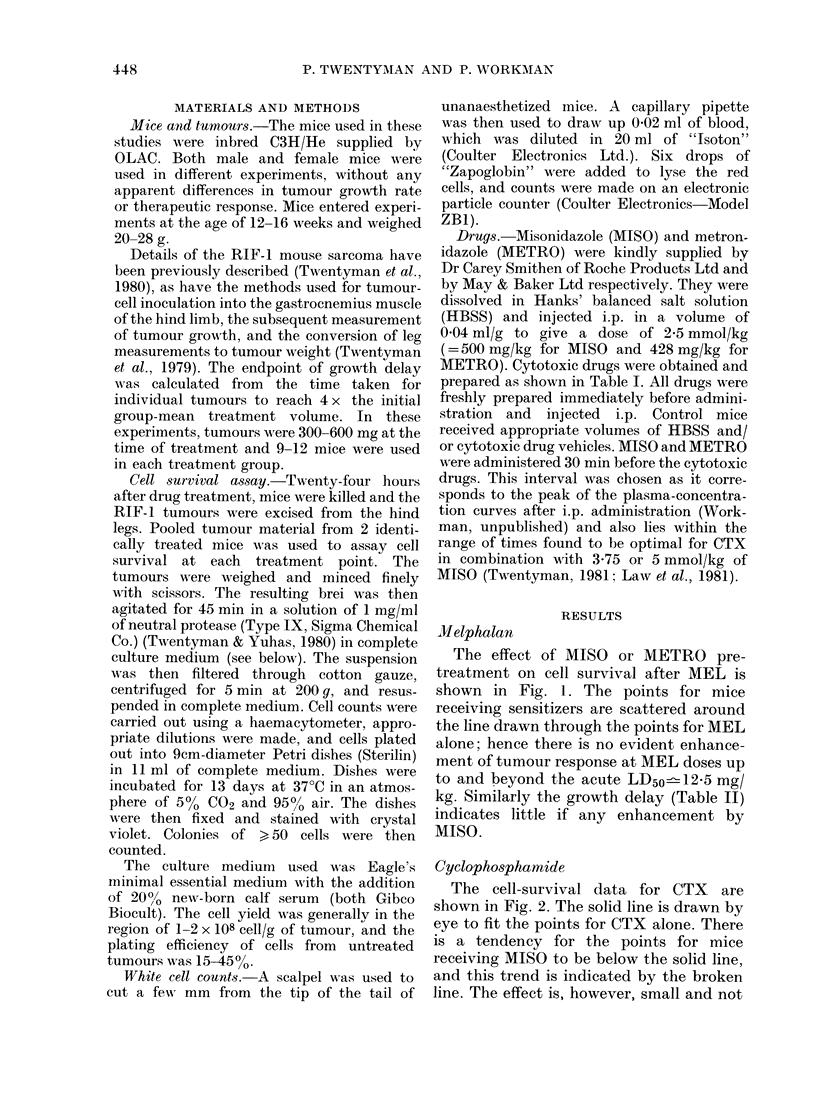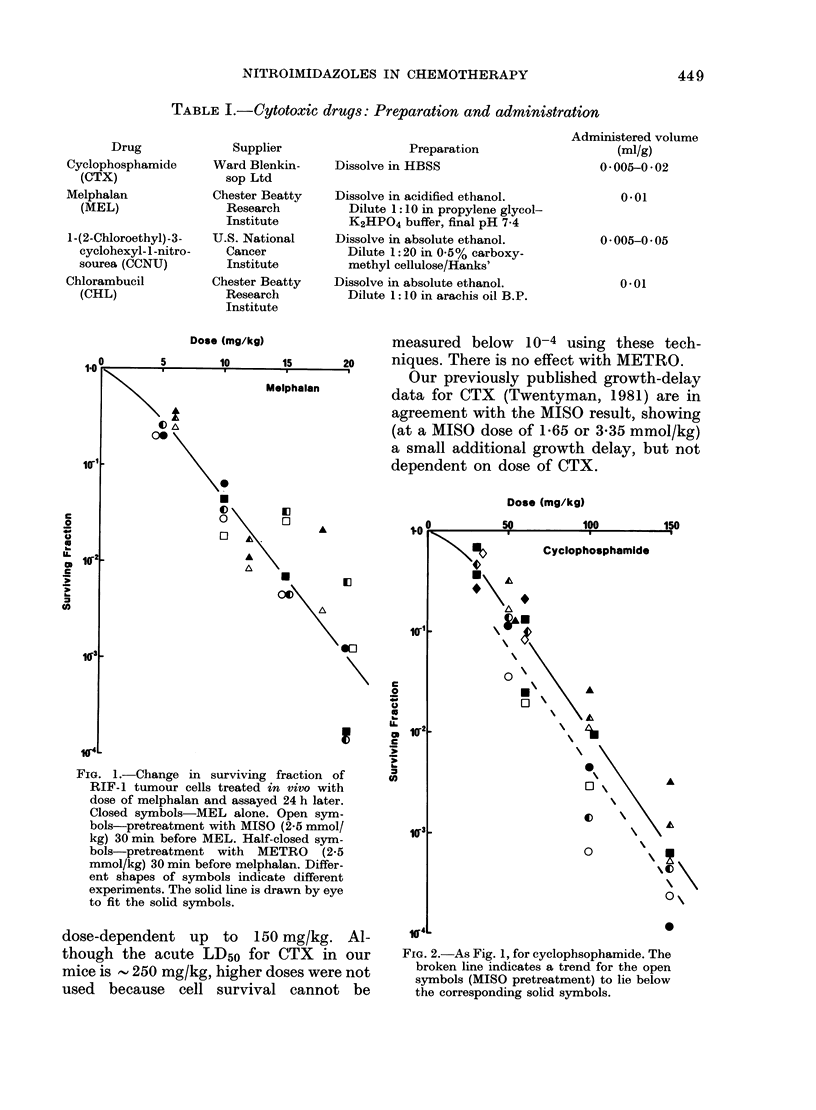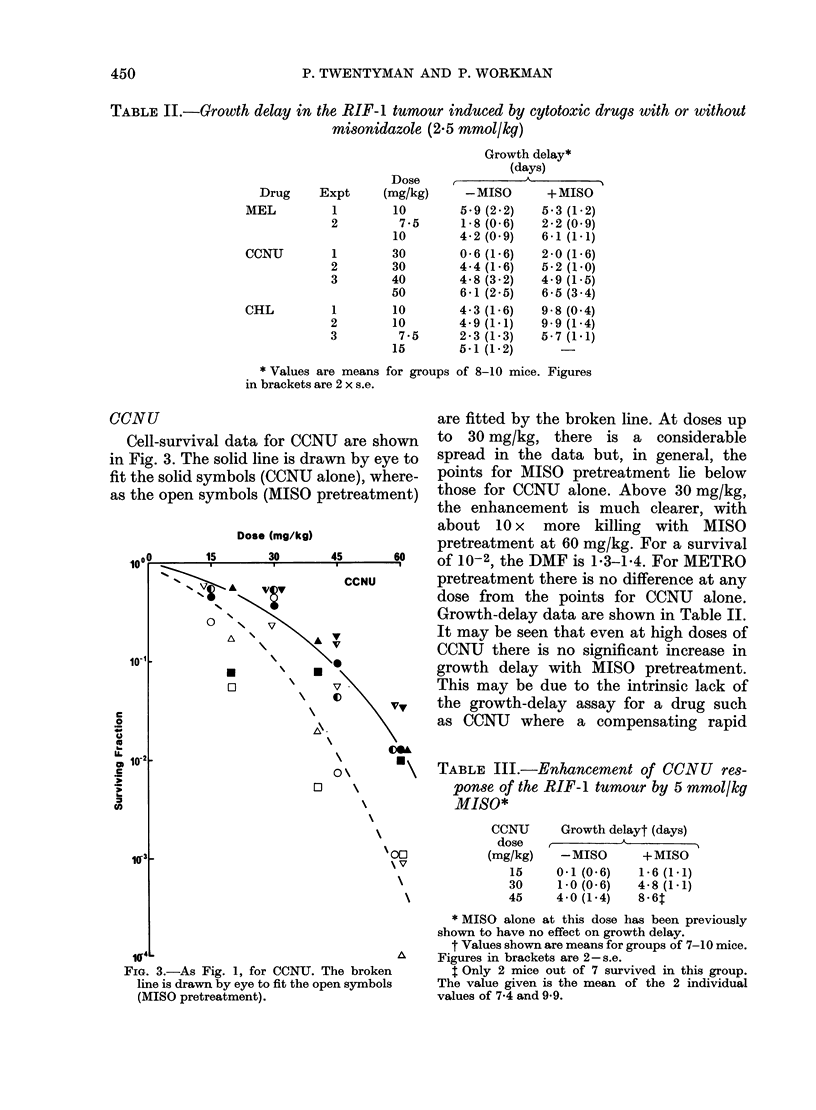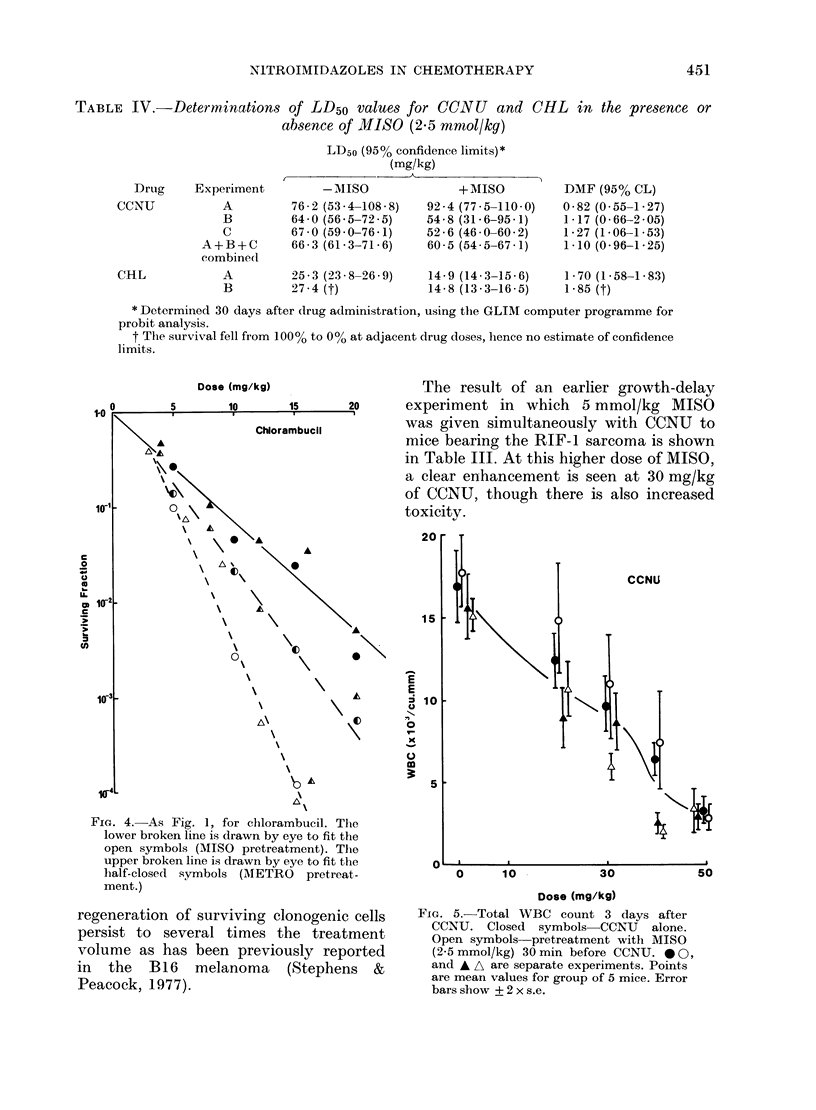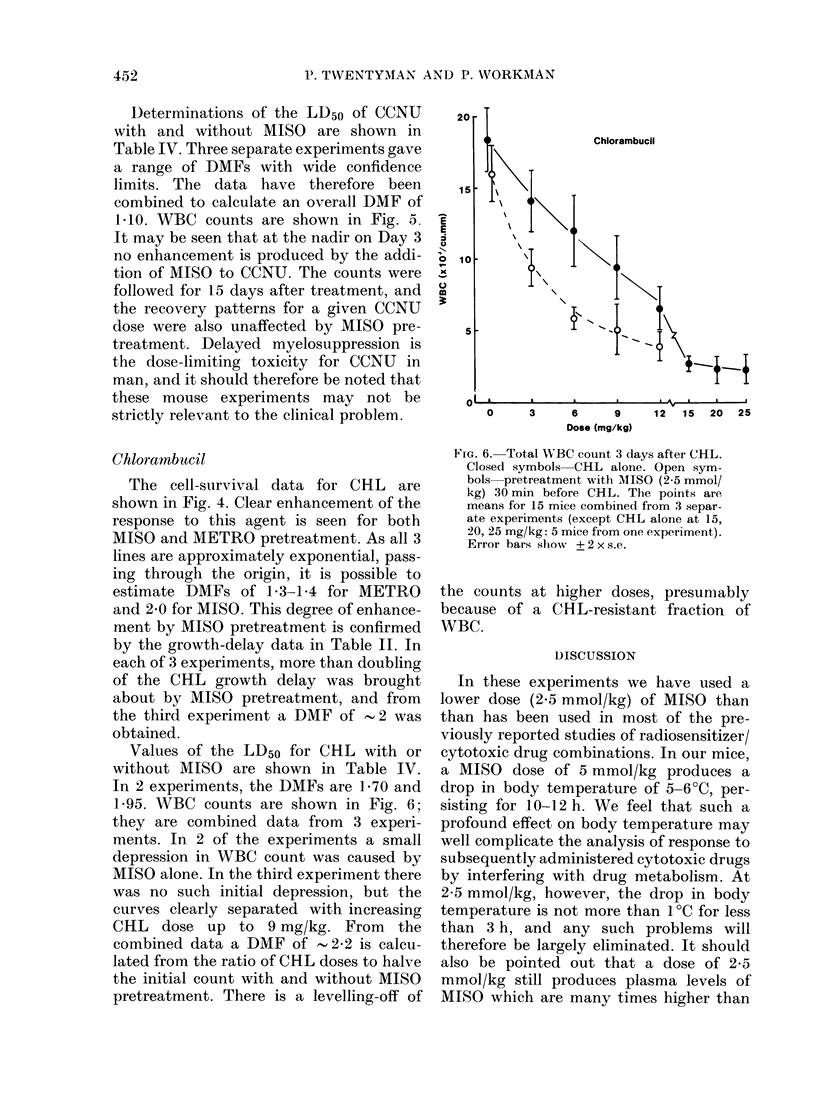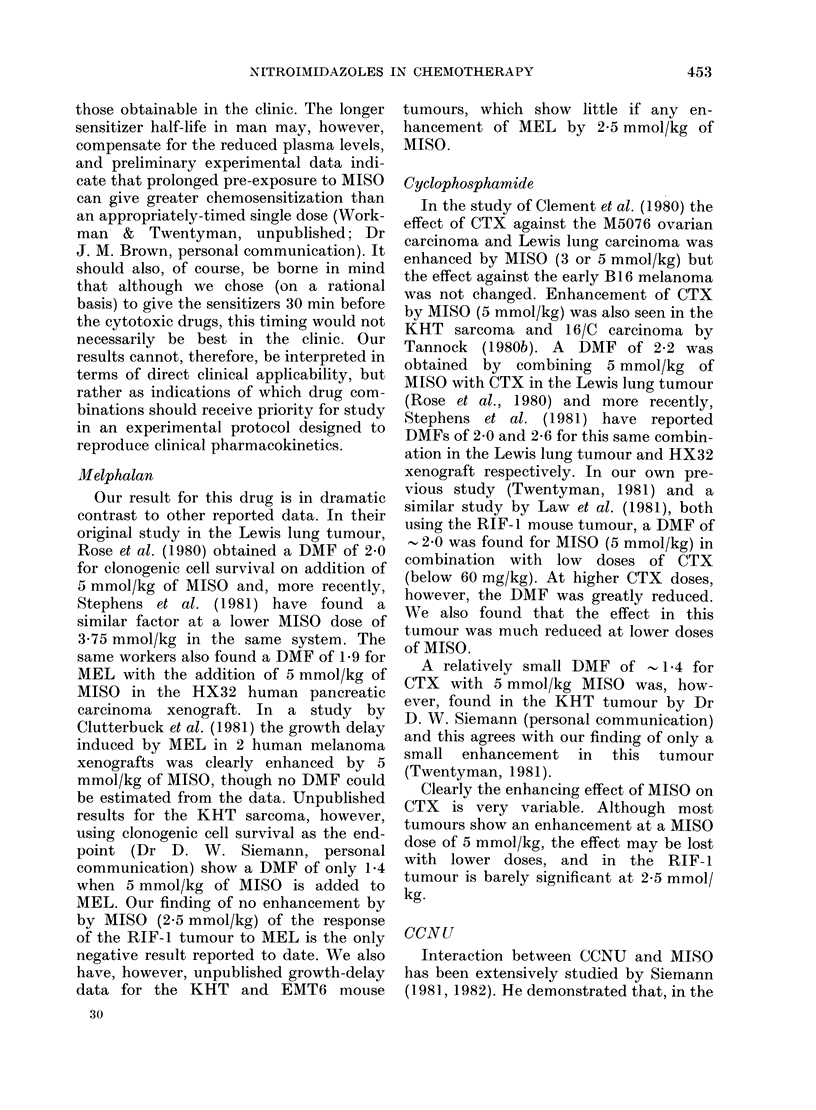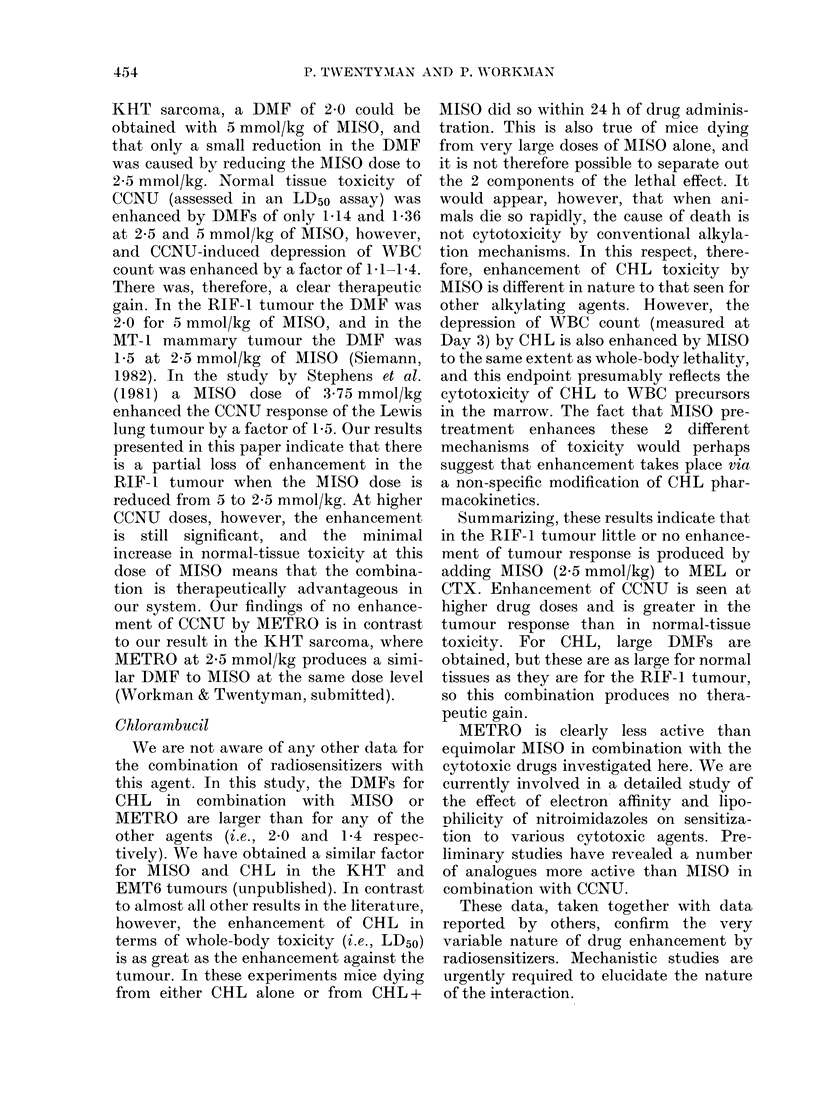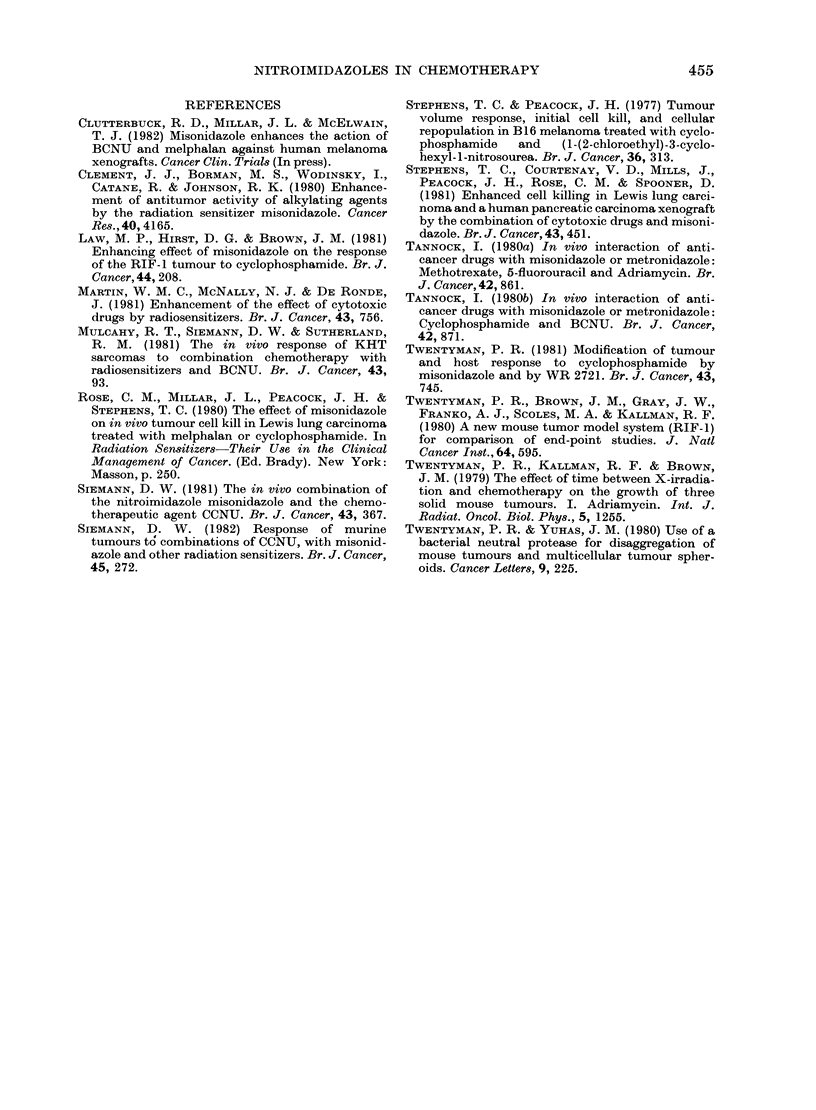# Effect of misonidazole or metronidazole pretreatment on the response of the RIF-1 mouse sarcoma to melphalan, cyclophosphamide, chlorambucil and CCNU.

**DOI:** 10.1038/bjc.1982.73

**Published:** 1982-03

**Authors:** P. Twentyman, P. Workman

## Abstract

The effect has been studied of adding either misonidazole (MISO) or metronidazole (METRO) to cytotoxic drug treatment of C3H mice bearing the RIF-1 sarcoma. The nitroimidazoles were injected 30 min before the cytotoxic drugs at a dose of 2 . 5 mmol/kg. Both clonogenic-cell survival and growth delay were measured as indicators of tumour response and depression in WBC count and acute lethality were used to indicate normal-tissue response. For melphalan, neither pretreatment agent produced any change in tumor response. For cyclophosphamide, no change was produced by METRO but a minimal increase in tumour response occurred with MISO. An enhancement of cell killing by CCNU was seen with MISO pretreatment, but there was no increase in tumour growth delay. METRO, however, did not enhance tumour response by either endpoint. WBC depression by CCNU was not enhanced by MISO pretreatment, and there was no significant reduction in the acute LD50. This indicates a therapeutic advantage from the addition of MISO to CCNU in this model system. For chlorambucil, considerable enhancement of tumour response followed either MISO or METRO pretreatment (dose-modifying factors of 2 . 0 and 1 . 4 respectively). However, the modification by MISO of normal-tissue response to chlorambucil was also enhanced by about a factor of 2, with no therapeutic gain.


*Br. J. Cancer* (1982) **45**, 447

### EFFECT OF MISONIDAZOLE OR METRONIDAZOLE PRETREATMENT ON THE RESPONSE OF THE RIF-1 MOUSE SARCOMA TO MELPHALAN, CYCLOPHOSPHAMIDE, CHLORAMBUCIL AND CCNU

P. TWENTYMAN AND P. WORKMAN

*From the M.R.C. Clinical Oncology and Radiotherapeutics Unit, Hills Road, Cambridge*

Received 18 August 1981 Accepted 9 November 1981

**Summary.—The effect has been studied of adding either misonidazole (MISO) or metronidazole (METRO) to cytotoxic drug treatment of C3H mice bearing the RIF-1 sarcoma. The nitroimidazoles were injected 30 min before the cytotoxic drugs at a dose of 2·5 mmol/kg. Both clonogenic-cell survival and growth delay were measured as indicators of tumour response and depression in WBC count and acute lethality were used to indicate normal-tissue response. For melphalan, neither pretreatment agent produced any change in tumour response. For cyclophosphamide, no change was produced by METRO but a minimal increase in tumour response occurred with MISO. An enhancement of cell killing by CCNU was seen with MISO pretreatment, but there was no increase in tumour growth delay. METRO, however, did not enhance tumour response by either endpoint. WBC depression by CCNU was not enhanced by MISO pretreatment, and there was no significant reduction in the acute $LD_{50}$. This indicates a therapeutic advantage from the addition of MISO to CCNU in this model system. For chlorambucil, considerable enhancement of tumour response followed either MISO or METRO pretreatment (dose-modifying factors of 2·0 and 1·4 respectively). However, the modification by MISO of normal-tissue response to chlorambucil was also enhanced by about a factor of 2, with no therapeutic gain.**

SEVERAL RECENT STUDIES have demonstrated that tumour response to cytotoxic drugs in mice can be enhanced by the 2-nitroimidazole misonidazole (MISO), a radiosensitizer of hypoxic cells (Rose *et al.*, 1980; Clement *et al.*, 1980; Tannock, 1980*a*, *b*; Siemann, 1981, 1982; Twentyman, 1981; Law *et al.*, 1981; Mulcahy *et al.*, 1981; Martin *et al.*, 1981; Clutterbuck *et al.*, 1982; Stephens *et al.*, 1981). In some of these studies (but not all) a therapeutic gain is claimed, in that enhancement of tumour response is greater than enhancement of normal-tissue damage.

Using the RIF-1 sarcoma in C3H mice, we have recently found that tumour growth delay induced by cyclophosphamide is considerably enhanced by the simultaneous administration of 5 mmol/kg (1 mg/g) MISO. At lower doses of MISO, however, much of the effect is lost (Twentyman, 1981).

In this paper, we describe experiments using the RIF-1 sarcoma in which we have studied the enhancement of a number of cytotoxic drugs by both MISO and the less electron-affinic 5-nitroimidazole analogue, metronidazole (METRO). The cytotoxic drugs studied were the nitrogen mustards, cyclophosphamide (CTX), melphalan (MEL) and chlorambucil (CHL), and the nitrosourea CCNU. Both growth delay and survival of clonogenic cells have been used as measures of tumour response. Depression of white blood cell (WBC) count and lethality have also been studied as indicators of normal-tissue response.

### MATERIALS AND METHODS

*Mice and tumours.*—The mice used in these studies were inbred C3H/He supplied by OLAC. Both male and female mice were used in different experiments, without any apparent differences in tumour growth rate or therapeutic response. Mice entered experiments at the age of 12–16 weeks and weighed 20–28 g.

Details of the RIF-1 mouse sarcoma have been previously described (Twentyman *et al.*, 1980), as have the methods used for tumour-cell inoculation into the gastrocnemius muscle of the hind limb, the subsequent measurement of tumour growth, and the conversion of leg measurements to tumour weight (Twentyman *et al.*, 1979). The endpoint of growth delay was calculated from the time taken for individual tumours to reach 4 × the initial group-mean treatment volume. In these experiments, tumours were 300–600 mg at the time of treatment and 9–12 mice were used in each treatment group.

*Cell survival assay.*—Twenty-four hours after drug treatment, mice were killed and the RIF-1 tumours were excised from the hind legs. Pooled tumour material from 2 identically treated mice was used to assay cell survival at each treatment point. The tumours were weighed and minced finely with scissors. The resulting brei was then agitated for 45 min in a solution of 1 mg/ml of neutral protease (Type IX, Sigma Chemical Co.) (Twentyman & Yuhas, 1980) in complete culture medium (see below). The suspension was then filtered through cotton gauze, centrifuged for 5 min at 200 *g*, and resuspended in complete medium. Cell counts were carried out using a haemacytometer, appropriate dilutions were made, and cells plated out into 9cm-diameter Petri dishes (Sterilin) in 11 ml of complete medium. Dishes were incubated for 13 days at 37°C in an atmosphere of 5% $CO_2$ and 95% air. The dishes were then fixed and stained with crystal violet. Colonies of ≥50 cells were then counted.

The culture medium used was Eagle's minimal essential medium with the addition of 20% new-born calf serum (both Gibco Biocult). The cell yield was generally in the region of $1–2 \times 10^8$ cell/g of tumour, and the plating efficiency of cells from untreated tumours was 15–45%.

*White cell counts.*—A scalpel was used to cut a few mm from the tip of the tail of unanaesthetized mice. A capillary pipette was then used to draw up 0·02 ml of blood, which was diluted in 20 ml of "Isoton" (Coulter Electronics Ltd.). Six drops of "Zapoglobin" were added to lyse the red cells, and counts were made on an electronic particle counter (Coulter Electronics—Model ZB1).

*Drugs.*—Misonidazole (MISO) and metronidazole (METRO) were kindly supplied by Dr Carey Smithen of Roche Products Ltd and by May & Baker Ltd respectively. They were dissolved in Hanks' balanced salt solution (HBSS) and injected i.p. in a volume of 0·04 ml/g to give a dose of 2·5 mmol/kg (=500 mg/kg for MISO and 428 mg/kg for METRO). Cytotoxic drugs were obtained and prepared as shown in Table I. All drugs were freshly prepared immediately before administration and injected i.p. Control mice received appropriate volumes of HBSS and/or cytotoxic drug vehicles. MISO and METRO were administered 30 min before the cytotoxic drugs. This interval was chosen as it corresponds to the peak of the plasma-concentration curves after i.p. administration (Workman, unpublished) and also lies within the range of times found to be optimal for CTX in combination with 3·75 or 5 mmol/kg of MISO (Twentyman, 1981; Law *et al.*, 1981).

### RESULTS

### *Melphalan*

The effect of MISO or METRO pretreatment on cell survival after MEL is shown in Fig. 1. The points for mice receiving sensitizers are scattered around the line drawn through the points for MEL alone; hence there is no evident enhancement of tumour response at MEL doses up to and beyond the acute $LD_{50} \simeq 12·5$ mg/kg. Similarly the growth delay (Table II) indicates little if any enhancement by MISO.

### *Cyclophosphamide*

The cell-survival data for CTX are shown in Fig. 2. The solid line is drawn by eye to fit the points for CTX alone. There is a tendency for the points for mice receiving MISO to be below the solid line, and this trend is indicated by the broken line. The effect is, however, small and not

TABLE I.—*Cytotoxic drugs: Preparation and administration*

| Drug | Supplier | Preparation | Administered volume (ml/g) |
|---|---|---|---|
| Cyclophosphamide (CTX) | Ward Blenkinsop Ltd | Dissolve in HBSS | 0·005–0·02 |
| Melphalan (MEL) | Chester Beatty Research Institute | Dissolve in acidified ethanol. Dilute 1:10 in propylene glycol–$K_2HPO_4$ buffer, final pH 7·4 | 0·01 |
| 1-(2-Chloroethyl)-3-cyclohexyl-1-nitrosourea (CCNU) | U.S. National Cancer Institute | Dissolve in absolute ethanol. Dilute 1:20 in 0·5% carboxymethyl cellulose/Hanks' | 0·005–0·05 |
| Chlorambucil (CHL) | Chester Beatty Research Institute | Dissolve in absolute ethanol. Dilute 1:10 in arachis oil B.P. | 0·01 |

FIG. 1.—Change in surviving fraction of RIF-1 tumour cells treated *in vivo* with dose of melphalan and assayed 24 h later. Closed symbols—MEL alone. Open symbols—pretreatment with MISO (2·5 mmol/kg) 30 min before MEL. Half-closed symbols—pretreatment with METRO (2·5 mmol/kg) 30 min before melphalan. Different shapes of symbols indicate different experiments. The solid line is drawn by eye to fit the solid symbols.

dose-dependent up to 150 mg/kg. Although the acute $LD_{50}$ for CTX in our mice is ~250 mg/kg, higher doses were not used because cell survival cannot be measured below $10^{-4}$ using these techniques. There is no effect with METRO.

Our previously published growth-delay data for CTX (Twentyman, 1981) are in agreement with the MISO result, showing (at a MISO dose of 1·65 or 3·35 mmol/kg) a small additional growth delay, but not dependent on dose of CTX.

FIG. 2.—As Fig. 1, for cyclophsophamide. The broken line indicates a trend for the open symbols (MISO pretreatment) to lie below the corresponding solid symbols.

TABLE II.—*Growth delay in the RIF-1 tumour induced by cytotoxic drugs with or without misonidazole (2·5 mmol/kg)*

| Drug | Expt | Dose (mg/kg) | Growth delay* (days) −MISO | +MISO |
|---|---|---|---|---|
| MEL | 1 | 10 | 5·9 (2·2) | 5·3 (1·2) |
| | 2 | 7·5 | 1·8 (0·6) | 2·2 (0·9) |
| | | 10 | 4·2 (0·9) | 6·1 (1·1) |
| CCNU | 1 | 30 | 0·6 (1·6) | 2·0 (1·6) |
| | 2 | 30 | 4·4 (1·6) | 5·2 (1·0) |
| | 3 | 40 | 4·8 (3·2) | 4·9 (1·5) |
| | | 50 | 6·1 (2·5) | 6·5 (3·4) |
| CHL | 1 | 10 | 4·3 (1·6) | 9·8 (0·4) |
| | 2 | 10 | 4·9 (1·1) | 9·9 (1·4) |
| | 3 | 7·5 | 2·3 (1·3) | 5·7 (1·1) |
| | | 15 | 5·1 (1·2) | — |

* Values are means for groups of 8–10 mice. Figures in brackets are 2 × s.e.

*CCNU*

Cell-survival data for CCNU are shown in Fig. 3. The solid line is drawn by eye to fit the solid symbols (CCNU alone), whereas the open symbols (MISO pretreatment) are fitted by the broken line. At doses up to 30 mg/kg, there is a considerable spread in the data but, in general, the points for MISO pretreatment lie below those for CCNU alone. Above 30 mg/kg, the enhancement is much clearer, with about 10× more killing with MISO pretreatment at 60 mg/kg. For a survival of $10^{-2}$, the DMF is 1·3–1·4. For METRO pretreatment there is no difference at any dose from the points for CCNU alone. Growth-delay data are shown in Table II. It may be seen that even at high doses of CCNU there is no significant increase in growth delay with MISO pretreatment. This may be due to the intrinsic lack of the growth-delay assay for a drug such as CCNU where a compensating rapid

FIG. 3.—As Fig. 1, for CCNU. The broken line is drawn by eye to fit the open symbols (MISO pretreatment).

TABLE III.—*Enhancement of CCNU response of the RIF-1 tumour by 5 mmol/kg MISO**

| CCNU dose (mg/kg) | Growth delay† (days) −MISO | +MISO |
|---|---|---|
| 15 | 0·1 (0·6) | 1·6 (1·1) |
| 30 | 1·0 (0·6) | 4·8 (1·1) |
| 45 | 4·0 (1·4) | 8·6‡ |

* MISO alone at this dose has been previously shown to have no effect on growth delay.

† Values shown are means for groups of 7–10 mice. Figures in brackets are 2 — s.e.

‡ Only 2 mice out of 7 survived in this group. The value given is the mean of the 2 individual values of 7·4 and 9·9.

TABLE IV.—*Determinations of $LD_{50}$ values for CCNU and CHL in the presence or absence of MISO (2·5 mmol/kg)*

| Drug | Experiment | LD50 (95% confidence limits)* (mg/kg) −MISO | +MISO | DMF (95% CL) |
|---|---|---|---|---|
| CCNU | A | 76·2 (53·4–108·8) | 92·4 (77·5–110·0) | 0·82 (0·55–1·27) |
| | B | 64·0 (56·5–72·5) | 54·8 (31·6–95·1) | 1·17 (0·66–2·05) |
| | C | 67·0 (59·0–76·1) | 52·6 (46·0–60·2) | 1·27 (1·06–1·53) |
| | A+B+C combined | 66·3 (61·3–71·6) | 60·5 (54·5–67·1) | 1·10 (0·96–1·25) |
| CHL | A | 25·3 (23·8–26·9) | 14·9 (14·3–15·6) | 1·70 (1·58–1·83) |
| | B | 27·4 (†) | 14·8 (13·3–16·5) | 1·85 (†) |

* Determined 30 days after drug administration, using the GLIM computer programme for probit analysis.

† The survival fell from 100% to 0% at adjacent drug doses, hence no estimate of confidence limits.

FIG. 4.—As Fig. 1, for chlorambucil. The lower broken line is drawn by eye to fit the open symbols (MISO pretreatment). The upper broken line is drawn by eye to fit the half-closed symbols (METRO pretreatment.)

regeneration of surviving clonogenic cells persist to several times the treatment volume as has been previously reported in the B16 melanoma (Stephens & Peacock, 1977).

The result of an earlier growth-delay experiment in which 5 mmol/kg MISO was given simultaneously with CCNU to mice bearing the RIF-1 sarcoma is shown in Table III. At this higher dose of MISO, a clear enhancement is seen at 30 mg/kg of CCNU, though there is also increased toxicity.

FIG. 5.—Total WBC count 3 days after CCNU. Closed symbols—CCNU alone. Open symbols—pretreatment with MISO (2·5 mmol/kg) 30 min before CCNU. ● ○, and ▲ △ are separate experiments. Points are mean values for group of 5 mice. Error bars show ± 2 × s.e.

Determinations of the $LD_{50}$ of CCNU with and without MISO are shown in Table IV. Three separate experiments gave a range of DMFs with wide confidence limits. The data have therefore been combined to calculate an overall DMF of 1·10. WBC counts are shown in Fig. 5. It may be seen that at the nadir on Day 3 no enhancement is produced by the addition of MISO to CCNU. The counts were followed for 15 days after treatment, and the recovery patterns for a given CCNU dose were also unaffected by MISO pretreatment. Delayed myelosuppression is the dose-limiting toxicity for CCNU in man, and it should therefore be noted that these mouse experiments may not be strictly relevant to the clinical problem.

*Chlorambucil*

The cell-survival data for CHL are shown in Fig. 4. Clear enhancement of the response to this agent is seen for both MISO and METRO pretreatment. As all 3 lines are approximately exponential, passing through the origin, it is possible to estimate DMFs of 1·3–1·4 for METRO and 2·0 for MISO. This degree of enhancement by MISO pretreatment is confirmed by the growth-delay data in Table II. In each of 3 experiments, more than doubling of the CHL growth delay was brought about by MISO pretreatment, and from the third experiment a DMF of ~2 was obtained.

Values of the $LD_{50}$ for CHL with or without MISO are shown in Table IV. In 2 experiments, the DMFs are 1·70 and 1·95. WBC counts are shown in Fig. 6; they are combined data from 3 experiments. In 2 of the experiments a small depression in WBC count was caused by MISO alone. In the third experiment there was no such initial depression, but the curves clearly separated with increasing CHL dose up to 9 mg/kg. From the combined data a DMF of ~2·2 is calculated from the ratio of CHL doses to halve the initial count with and without MISO pretreatment. There is a levelling-off of

FIG. 6.—Total WBC count 3 days after CHL. Closed symbols—CHL alone. Open symbols—pretreatment with MISO (2·5 mmol/kg) 30 min before CHL. The points are means for 15 mice combined from 3 separate experiments (except CHL alone at 15, 20, 25 mg/kg: 5 mice from one experiment). Error bars show ±2×s.e.

the counts at higher doses, presumably because of a CHL-resistant fraction of WBC.

### DISCUSSION

In these experiments we have used a lower dose (2·5 mmol/kg) of MISO than than has been used in most of the previously reported studies of radiosensitizer/cytotoxic drug combinations. In our mice, a MISO dose of 5 mmol/kg produces a drop in body temperature of 5–6°C, persisting for 10–12 h. We feel that such a profound effect on body temperature may well complicate the analysis of response to subsequently administered cytotoxic drugs by interfering with drug metabolism. At 2·5 mmol/kg, however, the drop in body temperature is not more than 1°C for less than 3 h, and any such problems will therefore be largely eliminated. It should also be pointed out that a dose of 2·5 mmol/kg still produces plasma levels of MISO which are many times higher than those obtainable in the clinic. The longer sensitizer half-life in man may, however, compensate for the reduced plasma levels, and preliminary experimental data indicate that prolonged pre-exposure to MISO can give greater chemosensitization than an appropriately-timed single dose (Workman & Twentyman, unpublished; Dr J. M. Brown, personal communication). It should also, of course, be borne in mind that although we chose (on a rational basis) to give the sensitizers 30 min before the cytotoxic drugs, this timing would not necessarily be best in the clinic. Our results cannot, therefore, be interpreted in terms of direct clinical applicability, but rather as indications of which drug combinations should receive priority for study in an experimental protocol designed to reproduce clinical pharmacokinetics.

*Melphalan*

Our result for this drug is in dramatic contrast to other reported data. In their original study in the Lewis lung tumour, Rose *et al.* (1980) obtained a DMF of 2·0 for clonogenic cell survival on addition of 5 mmol/kg of MISO and, more recently, Stephens *et al.* (1981) have found a similar factor at a lower MISO dose of 3·75 mmol/kg in the same system. The same workers also found a DMF of 1·9 for MEL with the addition of 5 mmol/kg of MISO in the HX32 human pancreatic carcinoma xenograft. In a study by Clutterbuck *et al.* (1981) the growth delay induced by MEL in 2 human melanoma xenografts was clearly enhanced by 5 mmol/kg of MISO, though no DMF could be estimated from the data. Unpublished results for the KHT sarcoma, however, using clonogenic cell survival as the end-point (Dr D. W. Siemann, personal communication) show a DMF of only 1·4 when 5 mmol/kg of MISO is added to MEL. Our finding of no enhancement by by MISO (2·5 mmol/kg) of the response of the RIF-1 tumour to MEL is the only negative result reported to date. We also have, however, unpublished growth-delay data for the KHT and EMT6 mouse tumours, which show little if any enhancement of MEL by 2·5 mmol/kg of MISO.

*Cyclophosphamide*

In the study of Clement *et al.* (1980) the effect of CTX against the M5076 ovarian carcinoma and Lewis lung carcinoma was enhanced by MISO (3 or 5 mmol/kg) but the effect against the early B16 melanoma was not changed. Enhancement of CTX by MISO (5 mmol/kg) was also seen in the KHT sarcoma and 16/C carcinoma by Tannock (1980*b*). A DMF of 2·2 was obtained by combining 5 mmol/kg of MISO with CTX in the Lewis lung tumour (Rose *et al.*, 1980) and more recently, Stephens *et al.* (1981) have reported DMFs of 2·0 and 2·6 for this same combination in the Lewis lung tumour and HX32 xenograft respectively. In our own previous study (Twentyman, 1981) and a similar study by Law *et al.* (1981), both using the RIF-1 mouse tumour, a DMF of $\sim$2·0 was found for MISO (5 mmol/kg) in combination with low doses of CTX (below 60 mg/kg). At higher CTX doses, however, the DMF was greatly reduced. We also found that the effect in this tumour was much reduced at lower doses of MISO.

A relatively small DMF of $\sim$1·4 for CTX with 5 mmol/kg MISO was, however, found in the KHT tumour by Dr D. W. Siemann (personal communication) and this agrees with our finding of only a small enhancement in this tumour (Twentyman, 1981).

Clearly the enhancing effect of MISO on CTX is very variable. Although most tumours show an enhancement at a MISO dose of 5 mmol/kg, the effect may be lost with lower doses, and in the RIF-1 tumour is barely significant at 2·5 mmol/kg.

*CCNU*

Interaction between CCNU and MISO has been extensively studied by Siemann (1981, 1982). He demonstrated that, in the

KHT sarcoma, a DMF of 2·0 could be obtained with 5 mmol/kg of MISO, and that only a small reduction in the DMF was caused by reducing the MISO dose to 2·5 mmol/kg. Normal tissue toxicity of CCNU (assessed in an $LD_{50}$ assay) was enhanced by DMFs of only 1·14 and 1·36 at 2·5 and 5 mmol/kg of MISO, however, and CCNU-induced depression of WBC count was enhanced by a factor of 1·1–1·4. There was, therefore, a clear therapeutic gain. In the RIF-1 tumour the DMF was 2·0 for 5 mmol/kg of MISO, and in the MT-1 mammary tumour the DMF was 1·5 at 2·5 mmol/kg of MISO (Siemann, 1982). In the study by Stephens *et al.* (1981) a MISO dose of 3·75 mmol/kg enhanced the CCNU response of the Lewis lung tumour by a factor of 1·5. Our results presented in this paper indicate that there is a partial loss of enhancement in the RIF-1 tumour when the MISO dose is reduced from 5 to 2·5 mmol/kg. At higher CCNU doses, however, the enhancement is still significant, and the minimal increase in normal-tissue toxicity at this dose of MISO means that the combination is therapeutically advantageous in our system. Our findings of no enhancement of CCNU by METRO is in contrast to our result in the KHT sarcoma, where METRO at 2·5 mmol/kg produces a similar DMF to MISO at the same dose level (Workman & Twentyman, submitted).

*Chlorambucil*

We are not aware of any other data for the combination of radiosensitizers with this agent. In this study, the DMFs for CHL in combination with MISO or METRO are larger than for any of the other agents (*i.e.*, 2·0 and 1·4 respectively). We have obtained a similar factor for MISO and CHL in the KHT and EMT6 tumours (unpublished). In contrast to almost all other results in the literature, however, the enhancement of CHL in terms of whole-body toxicity (*i.e.*, $LD_{50}$) is as great as the enhancement against the tumour. In these experiments mice dying from either CHL alone or from CHL+MISO did so within 24 h of drug administration. This is also true of mice dying from very large doses of MISO alone, and it is not therefore possible to separate out the 2 components of the lethal effect. It would appear, however, that when animals die so rapidly, the cause of death is not cytotoxicity by conventional alkylation mechanisms. In this respect, therefore, enhancement of CHL toxicity by MISO is different in nature to that seen for other alkylating agents. However, the depression of WBC count (measured at Day 3) by CHL is also enhanced by MISO to the same extent as whole-body lethality, and this endpoint presumably reflects the cytotoxicity of CHL to WBC precursors in the marrow. The fact that MISO pretreatment enhances these 2 different mechanisms of toxicity would perhaps suggest that enhancement takes place *via* a non-specific modification of CHL pharmacokinetics.

Summarizing, these results indicate that in the RIF-1 tumour little or no enhancement of tumour response is produced by adding MISO (2·5 mmol/kg) to MEL or CTX. Enhancement of CCNU is seen at higher drug doses and is greater in the tumour response than in normal-tissue toxicity. For CHL, large DMFs are obtained, but these are as large for normal tissues as they are for the RIF-1 tumour, so this combination produces no therapeutic gain.

METRO is clearly less active than equimolar MISO in combination with the cytotoxic drugs investigated here. We are currently involved in a detailed study of the effect of electron affinity and lipophilicity of nitroimidazoles on sensitization to various cytotoxic agents. Preliminary studies have revealed a number of analogues more active than MISO in combination with CCNU.

These data, taken together with data reported by others, confirm the very variable nature of drug enhancement by radiosensitizers. Mechanistic studies are urgently required to elucidate the nature of the interaction.

### REFERENCES

Clutterbuck, R. D., Millar, J. L. & McElwain, T. J. (1982) Misonidazole enhances the action of BCNU and melphalan against human melanoma xenografts. *Cancer Clin. Trials* (In press).

Clement, J. J., Borman, M. S., Wodinsky, I., Catane, R. & Johnson, R. K. (1980) Enhancement of antitumor activity of alkylating agents by the radiation sensitizer misonidazole. *Cancer Res.*, **40**, 4165.

Law, M. P., Hirst, D. G. & Brown, J. M. (1981) Enhancing effect of misonidazole on the response of the RIF-1 tumour to cyclophosphamide. *Br. J. Cancer*, **44**, 208.

Martin, W. M. C., McNally, N. J. & De Ronde, J. (1981) Enhancement of the effect of cytotoxic drugs by radiosensitizers. *Br. J. Cancer*, **43**, 756.

Mulcahy, R. T., Siemann, D. W. & Sutherland, R. M. (1981) The *in vivo* response of KHT sarcomas to combination chemotherapy with radiosensitizers and BCNU. *Br. J. Cancer*, **43**, 93.

Rose, C. M., Millar, J. L., Peacock, J. H. & Stephens, T. C. (1980) The effect of misonidazole on *in vivo* tumour cell kill in Lewis lung carcinoma treated with melphalan or cyclophosphamide. In *Radiation Sensitizers—Their Use in the Clinical Management of Cancer.* (Ed. Brady). New York: Masson, p. 250.

Siemann, D. W. (1981) The *in vivo* combination of the nitroimidazole misonidazole and the chemotherapeutic agent CCNU. *Br. J. Cancer*, **43**, 367.

Siemann, D. W. (1982) Response of murine tumours to combinations of CCNU, with misonidazole and other radiation sensitizers. *Br. J. Cancer*, **45**, 272.

Stephens, T. C. & Peacock, J. H. (1977) Tumour volume response, initial cell kill, and cellular repopulation in B16 melanoma treated with cyclophosphamide and (1-(2-chloroethyl)-3-cyclohexyl-1-nitrosourea. *Br. J. Cancer*, **36**, 313.

Stephens, T. C., Courtenay, V. D., Mills, J., Peacock, J. H., Rose, C. M. & Spooner, D. (1981) Enhanced cell killing in Lewis lung carcinoma and a human pancreatic carcinoma xenograft by the combination of cytotoxic drugs and misonidazole. *Br. J. Cancer*, **43**, 451.

Tannock, I. (1980*a*) *In vivo* interaction of anticancer drugs with misonidazole or metronidazole: Methotrexate, 5-fluorouracil and Adriamycin. *Br. J. Cancer*, **42**, 861.

Tannock, I. (1980*b*) *In vivo* interaction of anticancer drugs with misonidazole or metronidazole: Cyclophosphamide and BCNU. *Br. J. Cancer*, **42**, 871.

Twentyman, P. R. (1981) Modification of tumour and host response to cyclophosphamide by misonidazole and by WR 2721. *Br. J. Cancer*, **43**, 745.

Twentyman, P. R., Brown, J. M., Gray, J. W., Franko, A. J., Scoles, M. A. & Kallman, R. F. (1980) A new mouse tumor model system (RIF-1) for comparison of end-point studies. *J. Natl Cancer Inst.*, **64**, 595.

Twentyman, P. R., Kallman, R. F. & Brown, J. M. (1979) The effect of time between X-irradiation and chemotherapy on the growth of three solid mouse tumours. I. Adriamycin. *Int. J. Radiat. Oncol. Biol. Phys.*, **5**, 1255.

Twentyman, P. R. & Yuhas, J. M. (1980) Use of a bacterial neutral protease for disaggregation of mouse tumours and multicellular tumour spheroids. *Cancer Letters*, **9**, 225.